# The impact of masculinity and femininity on disordered eating symptoms and the mediating role of muscularity ideals

**DOI:** 10.1038/s41598-025-19246-6

**Published:** 2025-09-17

**Authors:** Gerrit Brandt, Marie Pahlenkemper, Vanessa C. Jürgensen, Martin S. Lehe, Nora M. Laskowski, Georg Halbeisen, Georgios Paslakis

**Affiliations:** Medical Faculty, University Clinic for Psychosomatic Medicine and Psychotherapy, Ruhr-University Bochum, Campus East-Westphalia, Virchowstr. 65, 32312 Luebbecke, Germany

**Keywords:** Gender roles, Masculinity, Femininity, Body ideals, Muscularity, Disordered eating, Muscle dysmorphia, Mediation analysis, Psychology, Psychiatric disorders

## Abstract

This study examines the role of gender role orientation in shaping body ideals and its impact on disordered eating and muscle dysmorphic symptoms in women. We explore how self-perceived Gender Roles regarding Masculinity and Femininity relate to thinness and muscularity ideals and whether these body ideals mediate the relationship between Gender Roles and disordered eating behaviors. A cross-sectional online survey was conducted with 304 adult women. Participants completed measures assessing gender role orientation, body ideals, disordered eating symptoms, and muscle dysmorphic symptoms. Mediation analyses were performed. Self-reported Masculinity was associated with stronger muscularity ideals, while Femininity predicted a weaker drive for muscularity. The mediation analysis showed that muscularity ideals completely mediated the relationship between self-perceived Masculinity or Femininity and disordered eating symptoms. The study highlights the importance of considering Gender Roles and muscularity ideals when examining the development of body image concerns and disordered eating behaviors. The findings suggest that promoting flexible gender role perceptions could be an effective focus for public health interventions aimed at preventing and reducing disordered eating. Future research should further investigate these relationships in clinical populations and explore additional mediators.

**Trial registration:** This study was preregistered on the Open Science Framework (OSF, https//osf.io/efn4v May 6, 2024).

## Background

What women consider to be their body ideal, i.e., a desired body shape, size, and composition, is a key determinant of body dissatisfaction, as body ideals establish the standards against which individuals compare their own physical appearance^1,2^. Body dissatisfaction has been associated with negative affect^3^, an increased risk for depression^4^, and most critically, with the development and maintenance of disordered eating^5^, Body Dysmorphia^6^, and Eating Disorders (EDs), including Anorexia Nervosa, Bulimia Nervosa, and Binge-Eating Disorder^7,8^. Since the treatment of mental health problems associated with body dissatisfaction is posing an increasing challenge to public healthcare systems^9^, it is important to understand which factors influence women’s current body ideals and how they link to different types of disordered eating.

Body ideals are influenced by the *zeitgeist* and are becoming increasingly diverse^10^. In the West, thinness has been idealized since the mid-20th century^11^, forming the basis of body-related concerns most commonly linked to the perception of being overweight^12^. Thin-ideal internalization in women has been directly associated with body dissatisfaction^1^ and has been identified as a predictor of ED onset^13^, as individuals strive to attain an often unobtainable ideal physique. Furthermore, research suggests that the internalization of a thin ideal frequently continues to be impactful during ED recovery and is a significant predictor of relapse risk^14,15^.

However, recent years saw a shift, among other trends, towards integrating thinness with athleticism^16^. Research suggests that, unlike men who often seek a more muscular and voluminous physique, women typically prioritize an athletic build characterized by defined muscles and low body fat^17^. These new beauty ideals combine a thin physique with increased muscularity, further narrowing beauty standards and creating additional challenges for women striving to meet them^18,19^. The internalization of the muscularity ideal in both men and women has been associated with dissatisfaction regarding their muscle tone and definition^20^. Additional evidence indicates that women’s drive for muscularity is strongly linked to harmful behaviors, including disordered eating and exercise dependence^21^. These behaviors are part of the symptom cluster of Muscle Dysmorphic Disorder^22^. Muscle Dysmorphic Disorder is a variant of Body Dysmorphia and is characterized by a compulsive preoccupation with muscularity and leanness as well as distorted body image^23^, which, however, often remains undetected^24^.

One hypothesized factor in shaping body ideals and thus leading to different forms of disordered eating behaviors among women is gender role orientation^25^. Gender role orientation refers to self-assessments of how closely one’s personality traits and behaviors align with traditional notions of the concepts of “Femininity” and “Masculinity”^26^. These masculine and feminine traits shape behavior and are linked to different beliefs and attitudes, including perceptions of one´s ideal body^27,28^. For instance, in Western cultures, Femininity has been associated with traits such as nurturance and emotional sensitivity, whereas Masculinity has been associated with assertiveness and independence^26,29^.

There is some indication that Gender Roles can pressure women to conform to specific female body ideals^30,31^, for instance, findings of an earlier meta-analysis indicated an overall small, heterogeneous positive relationship between Femininity and disordered eating^32^. Furthermore, women who exhibit characteristics associated with traditional Femininity, such as passivity, emotional dependence, and a strong desire for social approval, show a higher vulnerability to the development of EDs, compared to women scoring lower on Femininity^33^. In line with these findings, another empirical study suggests that women who identify strongly with traditional feminine roles are more likely to experience disordered eating behavior^34^. Mahalik et al.^35^ also found conformity to feminine norms positively related to ED psychopathology. However, Green, Davids, Skaggs, Riopel, & Hallengren^36^ only found one traditionally feminine characteristic (i.e., thinness orientation) to predict ED symptomatology using the same feminine norms measure as Mahalik et al.^35^. Furthermore, Behar et al.^37^ observed that women with less traditionally feminine and more “androgynous” traits were less prevalent among individuals with EDs compared to those without EDs. Yet, some studies question whether traditional Femininity increases the risk of developing EDs. For instance, a negative correlation was identified between Masculinity and scores on ED psychopathology^38^, while Femininity showed no significant association with unhealthy/disordered eating behavior^25^.

Research on masculine Gender Roles and disordered eating in women remains scarce. In one mixed gender sample, masculine gender role dimensions (i.e., unmitigated agency, male-typed behaviors and male sex-specific behaviors) were positively associated with the drive for muscularity in women^39^. Furthermore, undergraduate women with masculine gender traits exhibited higher levels of disordered eating compared to androgynous women^40^. Lampis et al.^41^ found that girls exhibiting higher levels of Masculinity were more prone to develop bulimia compared to those with lower Masculinity levels. Contrary to that, Reiter & Davis^42^ found Masculinity was a protective factor for women regarding disordered body image and eating behavior, and Blazek & Carter^43^ found that feeling more masculine predicted lower disordered eating behavior in adolescent girls.

The observed heterogeneity in findings regarding the influence of Gender Roles on ED symptoms may be attributable to several factors. For instance, a stronger identification with masculine ideals may also increase the drive for a more muscular physique, potentially leading to excessive strength training or restrictive eating in an effort to alter body composition behaviors often associated with Muscle Dysmorphic Disorder^34^. While Muscle Dysmorphic Disorder has traditionally been studied in men, its prevalence has increasingly risen among women^44,45^. To our knowledge, no detailed examination of this context exists to date but may contribute to the heterogeneity in study findings. Furthermore, differences in methodologies and sample characteristics across studies likely contribute to the inconsistencies. For example, measurement inconsistencies or limitations in the assessment of gender role orientations in previous research may have introduced systematic inaccuracies. Many scales have been designed, developed, and widely used for measuring traits traditionally considered as typically male vs. typically female. Most available tools classify individuals solely as either feminine or masculine, without allowing for the identification as both or neither. Early efforts to conceptualize Gender Roles framed Masculinity and Femininity as two poles of one dimension rooted in gender stereotypes allowing individuals to position themselves along this dimension. Another methodological limitation is the exclusive focus on Femininity in certain measures. For example, the Conformity to Feminine Norms Inventory (CFNI)^35^ is an 84-item self-report measure with eight subscales and a global score, assessing only women’s adherence to feminine norms within the dominant American culture. The Bem Sex Role Inventory (BSRI)^46^, however, measures the internalization of gender-stereotypic traits using 60 adjectives categorized as masculine, feminine, or neutral allowing individuals to score on these independent dimensions. This measure follows a progressive and more valid approach to assessing social gender role orientation. However, this measure, which continues to be widely used, was developed in earlier decades. Over time, shifting Gender Roles have likely reduced the ability to accurately reflect variations in how individuals ascribe stereotypical traits to themselves, today^47,48^.

Recently, Kachel, Steffens, & Niedlich^49^ developed the Traditional Masculinity and Femininity Scale (TMF) to address the limitations of earlier conceptualizations of Gender Roles, which framed Masculinity and Femininity as distinct dimensions rooted in gender stereotypes. This measure allows individuals to assess themselves in terms of Femininity and Masculinity independently, enabling identification as both, neither, or somewhere in between. Furthermore, the scale reflects enduring characteristics, including traits, appearances, interests, and behaviors historically associated with women and men, respectively. By extending on this understanding, the TMF scale provides a more nuanced approach by directly measuring Femininity and Masculinity along a two-dimensional spectrum to capture the proposed multidimensionality of Gender Roles.

Also, another reason for the heterogeneity of findings could be that traditional ED measures primarily focus on well-established disorders, potentially overlooking emerging or less commonly recognized conditions such as Avoidant/Restrictive Food Intake Disorder (ARFID)^50^ which is often associated with Orthorexia nervosa. Given the relatively limited research on this diagnosis, which is now classified as a feeding and eating disorder affecting individuals across the lifespan^51^, its distinct symptomatology may not be adequately captured by existing assessment tools.

## The present study

Previous studies have established a link between Gender Roles and both disordered eating behaviors and Muscle Dysmorphic Symptoms; however, findings have been heterogeneous, and the exact relationships, as well as potential confounding or mediating factors, remain unclear. Building on this previous research and utilizing a new reliable, valid, and bi-dimensional measure for traditional Gender Roles, this study examined the influence of Gender Role orientation on the relationship between body ideals, disordered eating, and Muscle Dysmorphic Symptoms.

Since previous measures have primarily assessed Gender Roles as a unidimensional construct, we anticipated that the two-dimensional approach would reveal opposing effect directions for Femininity and Masculinity. Furthermore, based on findings from the literature, we expected Femininity to predict thinness ideals and Masculinity to predict muscularity ideals. We also expected that Gender Roles would not only have a direct effect on ED symptoms and Muscle Dysmorphic Symptoms, but that body ideals would serve as a mediator. More specifically, we hypothesized that Femininity would influence ED symptoms through thinness ideals, while Masculinity would impact Muscle Dysmorphic Symptoms via muscularity ideals.

To investigate this, we conducted a survey study among women assessing Gender Roles, body ideals, ED symptoms (taking ARFID into account), and Muscle Dysmorphic Symptoms, analyzing the data using mediation analysis. To our knowledge, no study to date has employed the TMF scale to explore these relationships. Addressing this gap is crucial, as understanding how gender role orientation influences body ideals and disordered eating can provide valuable insights into the societal and psychological factors shaping disordered eating. Given the evolving nature of Gender Roles and societal norms, exploring these dynamics may provide a foundation for improving therapeutic approaches by better addressing the unique challenges faced by individuals with diverse gender identities and role orientations.

### Methods

#### Participants and procedures

The study examined disordered eating behavior in adult women (ages 18 and older) by analyzing data from a cross-sectional online survey. The project was pre-registered on the Open Science Framework (OSF, 10.17605/OSF.IO/EFN4V) and received ethics approval from our faculty´s ethics board (AZ 2022 − 910_1) on March 15, 2024. All study procedures adhered to the ethical standards of the Declaration of Helsinki and informed consent was obtained from all participants before the beginning of the study.

Based on the power estimation from previous studies conducted by our research group^52^, a total of 300 participants were targeted for recruitment. A two-pronged recruitment strategy was used to guarantee variety within the sample. On May 31, 2024, two-thirds of the participants were recruited via Prolific (https://www.prolific.com/), a widely used online platform for reliable and diverse participant recruitment. The remaining one-third of the sample data was collected through personal contacts, university mailing lists, and advertisements on the university website between May 8 and June 27, 2024. The survey was administered using jsPsych^53^. Participants were informed that the study focused on disordered eating among women. Those recruited via Prolific received compensation of £4.50, while university students in the convenience sample earned research participation credits.

## Measures

### Sociodemographic variables

Participants provided demographics, including gender (man, woman, diverse/intersex, or no answer), age, weight, height, and sexual orientation (coded non-heterosexual vs. heterosexual). Although only female individuals were eligible to participate via Prolific, we included multiple gender identity options to allow participants to self-identify in line with an inclusive understanding of gender. Following the German Federal Statistical Office´s updated definition^54^, participants were asked to indicate if they had a migration background (yes/no), based on the question “Were you or at least one of your parents born abroad?”. Moreover, marital status (single, married, divorced, or widowed), living situation (alone or with others), and education level (≤ 12 years or > 12 years) were recorded. Participants were asked open-ended questions about a potential history of EDs and if they were currently undergoing treatment. Additionally, participants were questioned whether they had previously participated in this study (yes/no). An attention check was included in the survey to ensure data integrity^55^.

## Gender roles

The Traditional Masculinity-Femininity Scale (TMF)^49^ was used to assess participants´ identification with feminine and masculine Gender Roles. The scale evaluated self-perceived Masculinity and Femininity by evaluating traits, interests, appearances, and behaviors traditionally associated with each gender. Masculinity and Femininity are each measured as individual dimensions in the TMF. Participants´ responses were scored on a four-item Likert scale that addresses the two core aspects of gender role-associated self-concept: gender role adaptation (how individuals currently perceive their gender role, e.g., “I consider myself as.”) and gender role preference (their interests, beliefs, and outward appearance concerning traditional Masculinity and Femininity, e.g., “Traditionally, my outer appearance would be considered.”). The response options ranging from 1 = not at all masculine/feminine to 4 = completely masculine/feminine.

Both scales demonstrated good internal consistency, with Cronbach’s α = 0.83 for the Masculinity scale and Cronbach’s α = 0.84 for the Femininity scale. The Gender Trait Difference was calculated as the difference between self-reported Masculinity and Femininity scores, with higher values indicating greater Masculinity relative to Femininity.

### Body ideals

To assess two dimensions of body image ideals in women, we used the Female Body Scale (FBS) and the Female Fit Body Scale (FFITBS)^56^. The FBS presents nine female body images ranging from emaciated to obese, whereas the FFTIBS shows nine images spanning from emaciated to highly muscular. For each series, participants were asked to select the image that most accurately reflected their current and ideal body shape. In the FFTIBS scale, ratings ranged from 0 (representing the most emaciated body) to 8 (indicating the most muscular), and in the FBS scale, from 0 (most obese) to 8 (most emaciated), with higher values reflecting a stronger Muscularity Ideal and Thinness Ideal, respectively.

## Disordered eating symptoms

We utilized the German version of the Eating Disorder Examination-Questionnaire (EDE-Q)^57^, which assesses behavioral and cognitive symptoms of disordered eating throughout the preceding 28 days. The questionnaire includes 22 attitudinal items (e.g., “Have you had a definite fear that you might gain weight”) along with six additional items assessing episodes of overeating, binge eating, binge days, and purging behavior (e.g., “How often during the past 28 days have you eaten an amount of food that others would regard as unusually large under similar circumstances?”), all rated on a seven-point scale from 0 (never) to 6 (every day). Since the factor structure of the EDE-Q has been questioned by previous research^58,59^, this study only used the global score, which demonstrated high internal consistency (Cronbach’s α = 0.90).

To evaluate ARFID symptoms, we adapted the Eating Disorders in Youth Questionnaire (EDY-Q)^60^. Originally developed for use in children and adolescents, the EDY-Q has recently been adapted for adults to allow broader applicability across age groups^61^. This questionnaire includes fourteen items addressing functional dysphagia (FD; e.g., “I am afraid of swallowing food”), selective eating (SE; e.g., “I am a picky eater”), food avoidance (FA; e.g., “If I was allowed, I would not eat”), and concerns related to low body weight. Additional questions on ARFID exclusion criteria such as pica, rumination, and weight/shape concerns were not included in the scale calculations. Participants´ responses were rated on a seven-point scale from 0 (never) to 6 (always). We report the total mean score of the EDY-Q, which showed a Cronbach’s α of 0.60. This moderate internal consistency can be explained by the FA subscale, which demonstrated low internal consistency (Cronbach’s α = 0.35), thereby contributing to the overall reliability of the total score. In addition, both the FD subscale (Cronbach’s α = 0.74) and the SE subscale (Cronbach’s α = 0.71) showed acceptable internal consistency within the sample.

Orthorexic eating behavior, characterized by an unhealthy obsession with healthy eating that may result in psychological, physical, and social impairments, was assessed using the ten item Duesseldorf Orthorexia Scale (DOS)^62^. Participants rated their responses to statements such as “It is more important to me to eat healthy food than to enjoy it” on a four-point scale ranging from 1 (“does not apply to me”) to 4 (“applies to me”). We report the total sum score. The scale demonstrated high internal consistency in our sample (Cronbach’s α = 0.87), which was comparable to the reliability reported by the original authors (α = 0.84).

## Muscle dysmorphic disorder

Moreover, we included the validated German version of the Muscle Dysmorphic Disorder Inventory (MDDI)^63^, which utilizes thirteen questions (e.g., “I think my legs are too thin”) scored on a five-point scale from 1 (never) to 5 (often) to evaluate muscularity-related body image concerns and behaviors. The questionnaire includes the three subscales drive for size (DS), appearance intolerance (AI), and functional impairment (FI), which all demonstrated high internal consistency (DS: Cronbach’s α = 0.80; AI: Cronbach’s α = 0.84; FI: Cronbach’s α = 0.84). We report the subscales as well as the total mean score, which had good internal consistency (Cronbach’s α = 0.77).

### Data analysis

Data analysis was performed using SPSS 30.0.0.0 (172)^64^ with the PROCESS macro version 4.2^65^ for mediation analyses. The figure was created using Python version 3.7.9^66^, utilizing the matplotlib package^67^ and networkx^68^.The reliability of the measures used in this study was evaluated by calculating Cronbach’s alpha (α). Cronbach’s α values above 0.70 were considered acceptable, with values over 0.80 indicating strong internal consistency^69^. Descriptive results are reported as means, SDs, or frequencies and percentages. Non-parametric Kendall’s Tau correlation coefficients were computed to examine the associations between the different assessment measures. The significance level for all inferential analyses was set at *p* ≤.05. Mediation analyses were performed to investigate the mediating roles of Muscularity and thinness ideals in the relationship between self-reported Masculinity and all outcomes, namely EDE-Q, EDY, and MDDI. A parallel mediation framework was utilized, allowing the simultaneous assessment of the mediating effects of Muscularity and thinness ideals in the relationship between self-reported Masculinity (and Femininity, respectively) and the EDE-Q global score, while controlling for age and sexual orientation by including them as a covariates in the model. This approach accounted for the unique contribution of each mediator while controlling for the influence of the other. For the mediation analysis, we utilized PROCESS v4.2 model four, employing a bootstrapped approach with 5,000 samples and a bias-corrected 95% confidence interval (CI). Statistical significance of the direct and indirect effects was assessed at the α = 0.05 level, including coefficients and p-values, were examined to interpret the magnitude and significance of these effects.

## Results

### Sample characteristics

A total of 304 individuals were initially recruited for the study. We excluded five multivariate outliers and eight participants who either did not disclose their gender or identified as non-binary. Consequently, the final sample comprised *n* = 291 participants included in the analysis. The mean age of the sample was 28.49 years (SD = 9.54, range 18–64) and the average body mass index (BMI) was 23.42 (SD = 5.10). In terms of sexual orientation, 66.3% (*n* = 193) reported exclusive attraction to the opposite gender, 22.3% (*n* = 65) reported attraction to men and women, and 7.6% (*n* = 22) reported exclusive attraction to the same gender, resulting in 29.9% (*n* = 87) indicating a non-heterosexual orientation. Furthermore, 29.9% (*n* = 87) of participants self-identified as having a migration background. Regarding educational attainment, 83.9% (*n* = 244) reported completing more than 12 years of schooling. With regard to relationship status, 77.7% (*n* = 226) indicated being single, 19.9% (*n* = 58) were married or in a committed partnership, and 2.4% (*n* = 7) reported being divorced. 72.9% (*n* = 212) of participants resided in shared households, whereas 26.8% (*n* = 78) reported living alone.

### Descriptive statistics and correlation matrix

The analysis revealed several noteworthy associations between gender traits and the psychological measures under study. Femininity and Masculinity were negatively correlated, indicating a tendency for these traits to be perceived or reported in contrast to one another within the sample. However, the strength of the correlation does not indicate perfect opposition, suggesting that Femininity and Masculinity coexist to varying degrees within individuals’ self-concept. Self-ascribed Femininity showed small negative correlations with the EDY mean total score and the MDDI mean total score. In contrast, Masculinity demonstrated weak positive associations with the EDY mean total score, the MDDI mean total score, and all three MDDI subscales: DS, AI, and FI.

The EDE-Q Global score was strongly positively correlated with the DOS total sum score, the MDDI mean total score, and the MDDI subscale AI. Additionally, the DOS total sum score was positively associated with the MDDI mean total score and its FI subscale.

Notably, neither Femininity nor Masculinity showed significant correlations with the EDE-Q Global score, the Thinness Ideal, or the Muscularity Ideal. The correlation matrix and descriptive statistics for the key measures and variables can be found in Table 1.

**Table 1:** Descriptive statistics and kendall’s Tau correlation matrix.

		Mean (SD, range)	1	2	3	4	5	6	7	8	9	10	11	12
1	TMF Femininity	12.67 (2.85, 4.00–18.00)	-											
2	TMF Masculinity	4.30 (2.95, 0.00–12.00)	− 0.660^**^	-										
3	TMF Gender Trait Difference^a^	−8.38 (5.47, −18.00–6.00)	− 0.845^**^	0.859^**^	-									
4	Thinnes Ideal	6.78 (0.73, 5.00–8.00)	− 0.006	− 0.018	− 0.001	-								
5	Muscularity Ideal	3.32 (0.91, 2.00–7.00)	− 0.089	0.091	0.087	− 0.569^**^	-							
6	EDE-Q Global score	1.75 (1.33, 0.00–5.57)	− 0.036	0.057	0.054	0.083	− 0.149^**^	-						
7	EDY Mean total score	1.26 (0.71, 0.00–3.60)	− 0.102^*^	0.104^*^	0.109^**^	0.138^**^	− 0.128^**^	0.064	-					
8	DOS total sum score	8.88 (5.45, 0.00–25.00)	− 0.027	0.002	0.017	0.132^**^	− 0.133^**^	0.392^**^	0.101^*^	-				
9	MDDI Mean total score	1.00 (0.55, 0.00–3.00)	− 0.105^*^	0.116^**^	0.115^**^	0.098^*^	− 0.008	0.448^**^	0.190^**^	0.380^**^	-			
10	MDDI Drive for Size	0.79 (0.74, 0.00–3.60)	− 0.085	0.097^*^	0.093^*^	0.094^*^	0.088	− 0.024	0.179^**^	0.125^**^	0.426^**^	-		
11	MDDI Appearance Intolerance	1.58 (1.01, 0.00–4.00)	− 0.083	0.091^*^	0.095^*^	0.007	− 0.073	0.626^**^	0.102^*^	0.266^**^	0.509^**^	− 0.023	-	
12	MDDI Functional Impairment	0.70 (0.79, 0.00–3.75)	− 0.051	0.103^*^	0.082	0.130^**^	0.003	0.247^**^	0.112^**^	0.407^**^	0.514^**^	0.215^**^	0.135^**^	-

Note. Two-sided; *correlation is significant at the 0.05 level; **correlation is significant at the 0.01 level. TMF = Traditional Masculinity-Femininity Scale^49^, EDE-Q = Eating Disorder Examination-Questionnaire^57^, EDY-Q = Eating Disorders in Youth-Questionnaire^60^, DOS = Duesseldorf Orthorexia Scale^62^, MDDI = Muscle Dysmorphic Disorder Inventory (Zeeck et al., 2018), ^a^Gender Trait Difference was calculated as the difference between self-reported Masculinity and Femininity scores, with higher values indicating greater Masculinity relative to Femininity while negative values indicating greater self-perceived Femininity than Masculinity.

### Mediation Analyses – Direct effects

#### Femininity

Regarding perceived Femininity and ED symptoms measured by the EDE-Q, we found no direct effect of Femininity (Table 2). However, avoidant-restrictive eating behaviors as measured by the EDY was predicted significantly by Femininity, in terms of higher feminine self-descriptions being associated with lower EDY scores. Femininity had no significant direct effect on the DOS sum score. Notably, Femininity had no direct effect on the Thinness ideal, which was contrary to expectations. One possible explanation for this finding is the low variance in thinness ideals scores within the sample, which was only 0.73. Responses on the 0 to 8 scale ranged exclusively between 5.00 and 8.00. Hence, low predictor variability may have reduced power and masked significant effects. However, perceived Femininity had a significant direct effect on muscularity ideals, with a stronger feminine self-description predicting a significantly lower drive for a muscular body ideal. Detailed statistics for the direct effects of Femininity on the mediator and outcome variables are presented in Table 2.

### Masculinity

Masculinity did not significantly predict EDE-Q global scores, indicating no direct association with overall ED symptoms (Table 2). However, it was a significant predictor of EDY scores, suggesting that higher self-perceived Masculinity was directly linked to increased avoidant-restrictive eating behaviors in our sample. No significant direct effect was observed for the DOS total score. In contrast, Masculinity showed a significant positive association with the MDDI mean score, indicating a direct link to higher levels of muscularity-oriented body image disturbance. Additionally, individuals with a stronger masculine self-description were more likely to endorse a muscular body ideal, while no significant association was found with thinness ideals. Table 2 summarizes these direct relationships between Masculinity and both the mediator and outcome variables.

### Gender trait difference

Further analyses revealed that a higher Gender Trait Difference—reflecting stronger self-identification as masculine relative to feminine—was significantly associated with increased avoidant-restrictive eating behaviors, as indicated by higher EDY-Q total mean scores. Additionally, individuals with a greater Gender Trait Difference reported significantly higher levels of Muscle Dysmorphic Symptoms, as measured by the MDDI total score, suggesting that a more masculine self-perception was linked to elevated muscularity-related body image concerns. A significant positive direct effect was also found between Gender Trait Difference and the endorsement of a muscularity ideal, indicating that participants who perceived themselves as more masculine than feminine were more likely to express a stronger preference for a muscular body ideal. A summary of how Gender Trait Difference directly influences the mediator and outcome variables is presented in Table 2.

**Table 2:** Direct effects of gender role dimensions on outcomes and mediators.

Predictor	Outcome/ Mediator	B	SE	t	*p*
TMF Femininity	EDE-Q Global score	−0.0139	0.0286	−0.4867	0.6269
	EDY Mean total score	−0.0442	0.0153	−2.879	**0.0043**
	DOS total sum score	−0.1131	0.1179	−0.9594	0.3382
	MDDI Mean total score	−0.0216	0.0118	−1.8215	0.0696
	Thinness Ideal	−0.0005	0.0157	−0.031	0.9753
	Muscularity Ideal	−0.0414	0.0197	−2.1062	**0.0361**
TMF Masculinity	EDE-Q Global score	0.0319	0.0272	1.1737	0.2415
	EDY Mean total score	0.0352	0.0147	2.4013	**0.017**
	DOS total sum score	0.1115	0.1121	0.9947	0.3208
	MDDI Mean total score	0.029	0.0112	2.5933	**0.01**
	Thinness Ideal	−0.0074	0.0149	−0.496	0.6203
	Muscularity Ideal	0.0452	0.0187	2.4241	**0.016**
TMF Gender Trait Difference^a^	EDE-Q Global score	0.0132	0.0148	0.8911	0.3736
	EDY Mean total score	0.0223	0.008	2.8036	**0.0054**
	DOS total sum score	0.0635	0.0611	1.0392	0.2996
	MDDI Mean total score	0.0144	0.0061	2.3573	**0.0191**
	Thinness Ideal	−0.0021	0.0081	−0.2537	0.7999
	Muscularity Ideal	0.0245	0.0102	2.4109	**0.0166**

Note. B = unstandardized regression coefficient; SE = standard error; t = t-value; p = p-value. Significant p-values (*p* <.05) are bolded. Controlled for age and sexual orientation. TMF = Traditional Masculinity-Femininity Scale^49^, EDE-Q = Eating Disorder Examination-Questionnaire^57^, EDY-Q = Eating Disorders in Youth-Questionnaire^60^, DOS = Duesseldorf Orthorexia Scale^62^, MDDI = Muscle Dysmorphic Disorder Inventory (Zeeck et al., 2018), ^a^Gender Trait Difference was calculated as the difference between self-reported Masculinity and Femininity scores, with higher values indicating greater Masculinity relative to Femininity while negative values indicating greater self-perceived Femininity than Masuclinity.

### Mediation

**Fig. 1:**
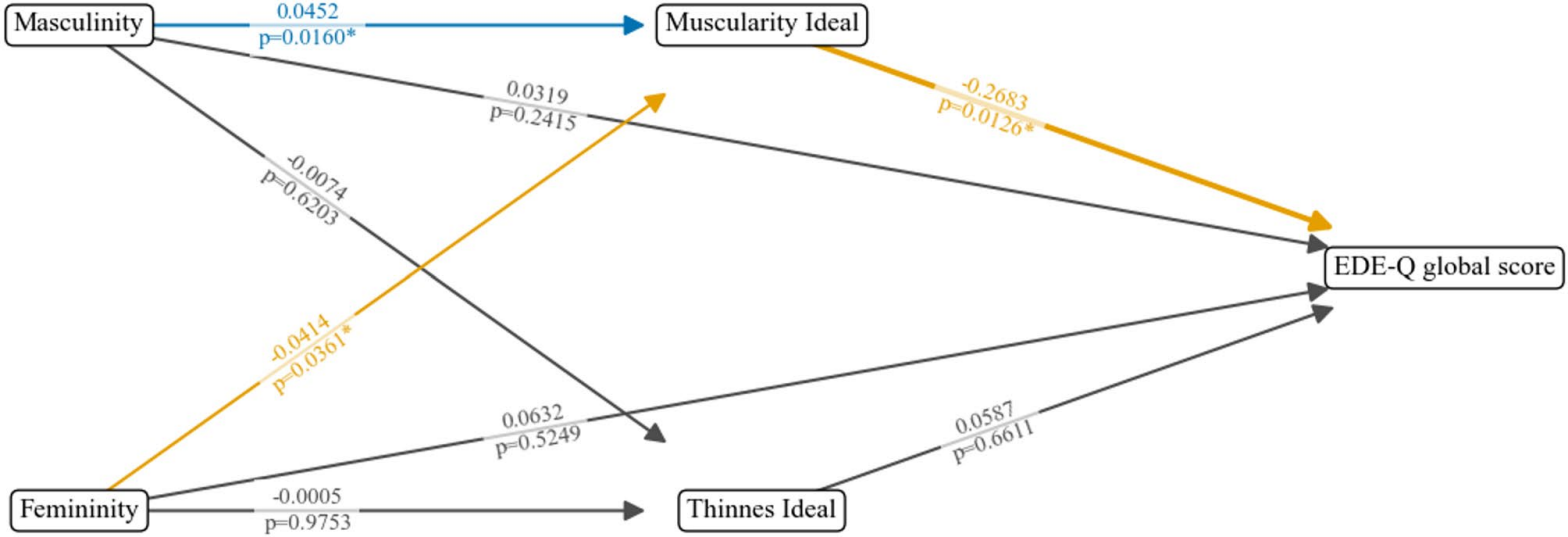
Path diagram of mediation analysis of the effect of Masculinity and Femininity on Eating Disorder Pathology mediated by Muscularity and Thinness Ideals. Analysis controlled for age and sexual orientation. *effect is significant at the 0.05 level; **effect is significant at the 0.01 level.; *TMF = Traditional Masculinity-Femininity Scale*^49^, *EDE-Q = Eating Disorder Examination-Questionnaire*^57^, *EDY-Q = Eating Disorders in Youth-Questionnaire*^60^, DOS = *Duesseldorf Orthorexia Scale*^62^, *MDDI = Muscle Dysmorphic Disorder Inventory (Zeeck et al.*,* 2018)*,* blue path = positive effect*,* orange path = negative effect*,* gray path = non-significant effect.*

Figure 1 illustrates the hypothesized relationships between the study variables using a path diagram. In this diagram, each node represents a variable, and the arrows indicate the proposed directions of influence between them. The thickness of the arrows corresponds to the magnitude of the effects. Color coding was applied to aid interpretation, with blue arrows indicating significant positive effects, orange indicating significant negative effects, and gray indicating non-significant effects.

As shown in the diagram and reported above, neither masculine nor feminine self-descriptions had a significant *direct* effect on global EDE-Q scores (ps > 0.24). However, the mediation analysis revealed meaningful *indirect* effects. Specifically, there was a significant positive association between self-perceived Masculinity and endorsement of muscularity ideals, while Femininity was significantly negatively associated with muscularity ideals. In turn, muscularity ideals had a significant negative effect on EDE-Q global scores.

These findings suggest that the impact of Masculinity and Femininity on ED symptoms is fully mediated through muscularity ideals - in other words, their effect on disordered eating symptoms occurs solely through their connection to internalized muscularity ideals.

The bootstrapped analysis provided evidence that Masculinity and Femininity influenced EDE-Q global scores *indirectly* through muscularity ideals (see Table 3). Once this indirect pathway was taken into account, no significant *direct* effects of either Masculinity or Femininity on disordered eating symptoms persisted.

**Table 3:** Results of the mediation analysis of EDE-Q global scores.

TMF Dimension	Direct Effect	Indirect Effect via Muscularity-Ideal	Indirect Effect via Thinnes Ideal	Total Indirect Effect
**Masculinity**	0.0319, *p* =.2415	**− 0.0121**, **95% CI = [−0.0283; − 0.0006]**	-,0004, 95% CI = [−0.0066; 0.0053]	− 0.0134, 95% CI = [−0.0288; − 0.0010]
**Femininity**	− 0.0139, *p* =.6269	**0.0106**, **95% CI = [0.0001; 0.0265]**	0.0000, 95% CI = [−0.0058; 0.0046]	0.0106, 95% CI = [−0.0031; 0.0266]

Notes. Results in bold are statistically significant; effects were considered statistically significant if the bootstrapped confidence interval did not include zero; EDE-Q = Eating Disorder Examination-Questionnaire^57^, TFM = Traditional Masculinity-Femininity Scale^49^; CI = confidence interval.

The mediation analyses conducted for Muscle Dysmorphic Symptoms (i.e., MDDI total score) and avoidant-restrictive eating behaviors (i.e., EDY total score) did not reveal any significant indirect effects via muscularity ideals or thinness ideals.

## Discussion

Gender Roles (i.e., social norms and stereotypes associated with Masculinity and Femininity) are enacted and adopted throughout life and have been shown to influence the risk of EDs^70^. Using the TMF, a newly validated and bi-dimensional measure of traditional Gender Roles, we found that Masculinity predicted stronger muscularity ideals, while Femininity predicted weaker drive for muscularity in a sample of women. Additionally, higher self-reported Masculinity was linked to increased avoidant-restrictive eating behaviors, and participants with higher Masculinity relative to Femininity scores were more likely to exhibit such eating patterns. However, neither masculine nor feminine self-description significantly influenced thinness ideals or EDE-Q global scores. Furthermore, our mediation analysis revealed a mediation effect, indicating that the influence of self-described Masculinity and Femininity on ED symptoms was explained through their effects on muscularity ideals.

Our results indicate that, as anticipated, the two-dimensional approach to measuring Gender Roles revealed opposing effect directions for Femininity and Masculinity on avoidant-restrictive eating behaviors and Muscle Dysmorphic Symptoms. Specifically, perceived Femininity had a reducing effect, while Masculinity had an amplifying effect. This may help explain previously inconsistent findings in the literature, as earlier studies often lacked a bidimensional and up-to-date assessment of Gender Roles^34,36,41,43^.

Self-ascribed Masculinity in this sample of women positively predicted muscularity ideals, as anticipated. Contrary to expectations, however, Femininity did not predict thinness ideals. This may be due to the restricted range of thinness ideal scores, which could have limited variability and reduced the power to detect significant effects. In other words, the women in this sample showed a highly uniform idealization of thinness. Femininity, however, was negatively associated with muscularity ideals. Furthermore, we found that Gender Roles were associated with EDE-Q scores indirectly through muscularity ideals, with higher Masculinity linked to greater endorsement of muscularity ideals, which in turn appeared to relate to lower disordered eating symptoms—suggesting a potentially protective role.

These findings indicate that muscularity ideals may play a meaningful role in the development of ED pathology. However, the negative association between muscularity ideals and EDE-Q scores, suggesting a potential protective effect, contradicts findings from other studies, which have reported positive (or null) associations between appearance ideals and disordered eating^8^. A possible explanation for this apparent discrepancy may lie in the design of the EDE-Q itself, which primarily assesses thinness-oriented eating pathology^59^. Individuals with higher muscularity ideals may shift their focus away from thinness toward muscularity-driven body goals. Such a shift may involve disordered behaviors, e.g., one-sided nutrition plans that are not calorically restricted and involve the consumption of (excessive) proteins and the avoidance of fats during “bulk” phases, accompanied by weight-lifting regimens, or the use of performance-enhancing substances^71^, all of which the EDE-Q does not effectively capture. As a result, lower EDE-Q scores in this group may reflect limitations of the assessment method rather than a true protective role of muscularity ideals.

Achieving a muscular physique is an increasingly prevalent concern among women^72^. The present study highlights that aspects of muscularity ideals may be important in the etiology of EDs. However, the EDE-Q - despite being considered the gold standard in ED assessment - does not include any items addressing muscularity-related concerns. There is a need for data-driven, validated diagnostic tools that capture the full spectrum of ED-relevant features in order to improve diagnostic accuracy and ensure timely access to appropriate support services.

Research indicates that disordered eating behaviors are prevalent in the general population and represent significant public health concerns. Between 2000 and 2018, the worldwide prevalence of EDs more than doubled, rising from 3.4 to 7.8%^73^. This increase is particularly alarming given that EDs have one of the highest mortality rates among psychiatric disorders^74^. Therefore, preventing EDs should be a priority within public health policies and initiatives. Considering diverse understandings of Gender Roles and variations in the internalization of gender ideals – particularly among women – may be valuable in a clinical context to support more individualized treatment approaches. On a broader scale, promoting flexible gender norms may support public health efforts to reduce socio-cultural risk factors associated with body dissatisfaction and disordered eating.

Furthermore, our findings suggest that fitness and muscularity ideals may be increasingly embedded in German society as muscularity ideals were prominent in our sample. This aligns with^75^, who highlight the growing prominence of muscularity in contemporary beauty standards. However, since our sample was not representative, these results cannot be generalized to the broader population with certainty.

In an earlier study, women with no discrepancy between their experienced and ideal gender perception reported fewer anorexic and bulimic symptoms, less concern with body shape, and higher self-esteem than those with a discrepancy^76^. However, further research is needed to explore discrepancies in gender identification in relation to a bi-dimensional measure of Gender Roles.

Previous research suggests that sexual orientation may influence body image and eating behaviors, likely mediated by distinct sociocultural pressures, minority stress, and divergent gender role expectations^77^. The minority stress theory^78,79^ posits that sexual and gender minorities who either experience or fear stigma and discrimination or have internalized homophobia are at increased risk for adverse mental health outcomes, including disordered eating^80^. A recent study reports elevated rates of eating pathology among sexual and gender minority youths and adults, which have indeed been linked to experiences of stigma and discrimination^81^. In the present study, sexual orientation was included as a control variable, and the observed mediation effects were not attributable to variance in sexual orientation. Nevertheless, sexual orientation may intersect with gender role internalization in ways that shape body image- and eating-related outcomes. Future studies with larger and more diverse samples should investigate how such intersections influence the internalization of gender roles, as this may be a pathway through which body image concerns and disordered eating develop in sexual and gender minorities.

### Limitations

Despite the strengths of this study, certain limitations must be acknowledged when interpreting the findings. It cannot be ruled out that additional factors influence the relationship between Gender Roles and ED pathology. Furthermore, we did not find any mediating effect of muscularity or thinness ideals on Muscle Dysmorphic Symptoms or avoidant-restrictive eating behaviors. This suggests that the proposed mediation model does not sufficiently explain the variance in these outcomes. Further studies should explore alternative mediators, including body dissatisfaction, perfectionism, and social comparison tendencies, and examine moderation effects to better understand the complex interplay between Gender Roles, body ideals, and disordered eating behaviors.

While this study found no significant association between Femininity and the Thinness Ideal, this finding should be interpreted with caution. The high endorsement of thinness in our sample may have resulted in a range restriction, limiting the statistical power to detect potential associations. Moreover, the FBS captures body ideals as a unidimensional construct, which may not reflect more nuanced aspects of thinness and muscularity ideals, such as the degree of internalization or variations in motivational or cultural aspects. Future studies should employ more nuanced and multidimensional measures of appearance ideals to better capture the complexity of these constructs and their associations.

Another limitation concerns the low reliability of the EDY-Q, particularly due to the low internal consistency observed in the FA subscale. While this may reduce the precision and interpretability of the findings, it may also mirror the fact that the underlying construct has not yet been fully established, highlighting the need for further conceptual clarification and empirical investigation.

In this study, we focused on women to establish clear initial effects. However, the generalizability to other groups, such as men, remains uncertain.

We used Prolific, which, despite its good metrics regarding data quality and participant diversity, still offers limited control over the quality of the data -similar to most online studies^82^.

This study examined body ideals as one important aspect of body dissatisfaction, which is widely understood to be a multifaceted construct^2^. While participants reported their body ideals, how they interpret or evaluate these ideals remains unclear. Additionally, the emotional and behavioral responses that may arise from perceived discrepancies between actual and ideal body are not yet well understood. Future research should explore these broader dimensions to gain a more nuanced understanding of body dissatisfaction and its relevance to ED pathology.

## Conclusion

This study confirms that Gender Roles influence body ideals and disordered eating behaviors. Masculinity was linked to stronger muscularity ideals and higher avoidant-restrictive eating behaviors, while Femininity predicted a weaker muscularity idealization. Mediation analysis showed that muscularity ideals fully explained the link between Gender Roles and ED symptoms. Promoting flexible gender role perceptions could be an effective focus for public health interventions aimed at preventing and reducing disordered eating. Future research should further examine muscularity ideals, particularly in representative samples as well as clinical populations, and explore alternative mediators to refine treatment strategies. Additionally, Gender Roles and muscularity ideals should be considered as important facets in the understanding and assessment of eating disorders.

## Data Availability

The datasets analyzed during the current study are available from the corresponding author on reasonable request.

## Abbreviations

ARFID: Avoidant/Restrictive Food Intake Disorder
BMI: Body Mass Index
BSRI: Bem Sex Role Inventory
CFNI: Conformity to Feminine Norms Inventory
CI: Confidence Interval
DOS: Duesseldorf Orthorexia Scale
DS: Drive for Size (subscale of MDDI)
EDE: Q–Eating Disorder Examination–Questionnaire
EDs: Eating Disorders
EDY: Q–Eating Disorders in Youth–Questionnaire
FA: Food Avoidance (subscale of EDY–Q)
FD: Functional Dysphagia (subscale of EDY–Q)
FBS: Female Body Scale
FFITBS: Female Fit Body Scale
FI: Functional Impairment (subscale of MDDI)
JS: JavaScript
MDDI: Muscle Dysmorphic Disorder Inventory
MDD: Muscle Dysmorphic Disorder
OSF: Open Science Framework
SE: Selective Eating (subscale of EDY–Q)
SD: Standard Deviation
SPSS: Statistical Package for the Social Sciences
TMF: Traditional Masculinity–Femininity Scale
